# Parke, Davis & Company’s Vaccine Virus

**Published:** 1902-01

**Authors:** 

**Affiliations:** Detroit


### CORRESPONDENCE.

### Parke, Davis & Company's Vaccine Virus.

*Editor Buffalo Medical Journal:*

Sir—We respectfully ask you to apprise your readers, on the faith of our positive assurance, that not one of the recent tetanus fatalities following vaccination at Camden, Atlantic City, Bristol, Brooklyn, Cleveland and St. John, N.B., succeeded the employment of our vaccine virus. In not a single, solitary one of these cases was our vaccine used. We incriminate no one's vaccine, but we propose to assert the truth about our own. If we can prevent it, no physician or pharmacist shall labor under the false impression that a fatality has ever followed, either by coincidence or by cause and effect, the application of vaccine virus or serum bearing our name. PARKE, DAVIS & CO.

Detroit, December 5, 1901.

